# Identification of an immunomodulatory lncRNA signature associated with immune cell reprogramming in high-grade glioma

**DOI:** 10.1038/s41417-025-00919-3

**Published:** 2025-06-17

**Authors:** Alessandro Canella, Prajwal Rajappa

**Affiliations:** 1 The Steve and Cindy Rasmussen Institute for Genomic Medicine, Nationwide Children’s Hospital, Columbus, OH USA; 2 Department of Pediatrics, The Ohio State University Wexner Medical Center, Columbus, OH USA; 3 Department of Neurological Surgery, The Ohio State University Wexner Medical Center, Columbus, OH USA

**Keywords:** Translational immunology, Cancer microenvironment, Paediatric cancer

## Abstract

High-grade gliomas (HGGs) are among the most aggressive brain tumors in pediatric, adolescent, and young adult (AYA) cancer patients, with a median survival of 12–15 months post-diagnosis. Their poor prognosis is driven by a highly immunosuppressive tumor immune microenvironment (TIME), which inhibits cytotoxic immune infiltration and anti-tumor response. This study investigated the involvement of long non-coding RNAs (lncRNAs) in shaping the immune phenotype of HGGs using two murine models: RCAS-PDGFb representing an immunosuppressive TME, and RCAS-BRAF V600E characterized by a signature more consistent with a pro-inflammatory TME. Transcriptomic analysis of tumor-infiltrating immune cells identified distinct lncRNA signatures associated with immunosuppressive and pro-inflammatory TMEs. Single-cell RNA sequencing and spatial transcriptomics supported context-dependent expression of these lncRNAs in high-grade glioma-associated immune cells, such as myeloid, T, and NK cells, and revealed their spatial distribution within the glioblastoma (GBM) TME. Several lncRNAs were enriched at the tumor edge and within necrotic regions in GBM patient samples, correlating with immunosuppression reprogramming and immune evasion mechanisms. These findings highlight specific immunomodulatory lncRNAs as potential players in the immunosuppressive glioma TME, and likely candidates for future studies aimed at developing novel therapeutic strategies to overcome immune suppression and improve clinical outcomes.

## Introduction

**Fig. 1:**
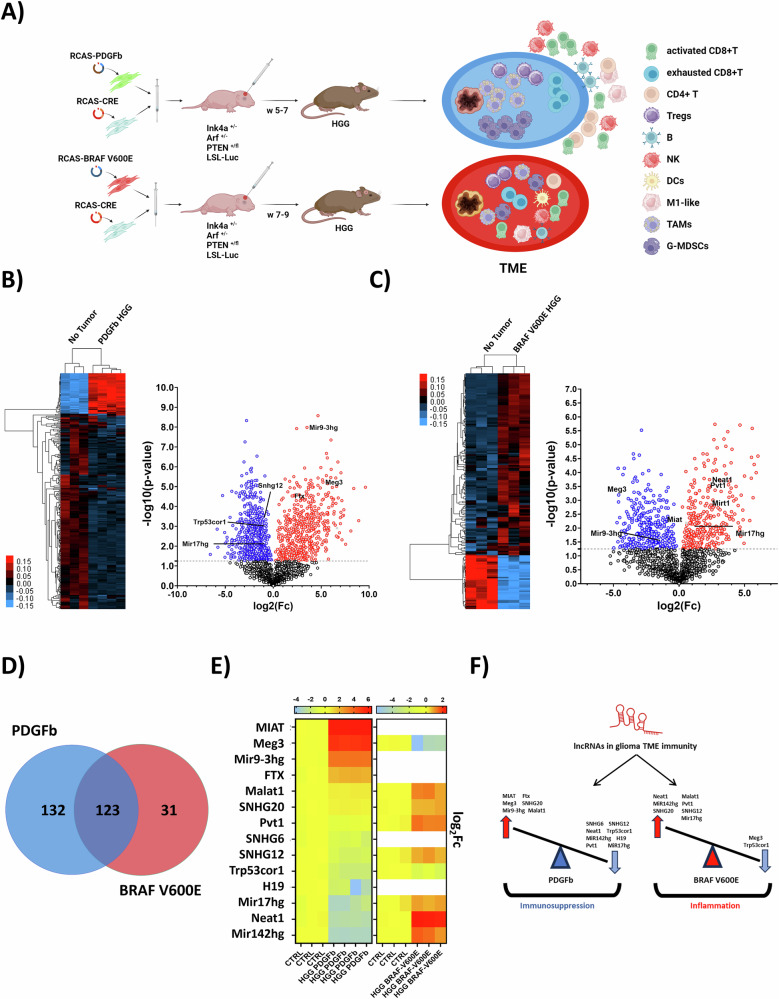
Expression of lncRNAs in the HGG TIME in RCAS/t-va immunosuppressive and pro-inflammatory models. **A** Schematic representation of the TIME in HGG RCAS murine models: RCAS-PDGFb develops a “cold” TIME, while RCAS-BRAF V600E develops a “hot” TIME, also characterized by infiltration of immunosuppressive myeloid cells and exhausted T cells (biorender.com). **B** Left: Dendrogram of the unsupervised hierarchical clustering analysis of total RNA sequencing in intratumoral immune cells from RCAS-PDGFb HGG mice (n = 4) compared with age-matched no-tumor controls (*n* = 3). Right: Volcano plot of differentially expressed lncRNAs in tumor-infiltrating immune cells (3665 genes in total). **C** Left: Dendrogram of the unsupervised hierarchical clustering analysis of total RNA sequencing in intratumoral immune cells from RCAS-BRAF V600E HGG mice (*n* = 3) compared with age-matched no-tumor controls (*n* = 3). Right: Volcano plot of differentially expressed lncRNAs in tumor-infiltrating immune cells (2819 genes in total). **B**, **C** Genes significantly upregulated are highlighted in , and those significantly downregulated are in . **D** Venn diagram showing the overlap of differentially expressed lncRNAs in the TIME of the HGG RCAS-PDGFb and HGG RCAS-BRAF V600E models. Numbers indicate lncRNAs unique to each model or shared between them. **E** Heatmaps of the immunoregulatory lncRNAs differentially expressed in tumor-infiltrating immune cells compared with no-tumor controls. **F** Hypothesis model illustrating the proposed role of lncRNAs in regulating the TIME (biorender.com). Statistical significance was calculated using an unpaired two-tailed Student’s *t*-test (*P* < 0.05).

In pediatric (<15 years old), adolescent and young adult (AYA, 15–39 years old) cancer patients, High-Grade Gliomas (HGGs) are among the most aggressive and lethal brain tumors [1]. HGGs account approximately 8–12% of brain tumors in pediatric patients and 30–40% in AYAs, with a poor prognosis and a dismal median survival of approximately 12–15 months post-diagnosis [2]. Despite advancements in therapies, high mortality persists due to the tumor’s mutational burden, angiogenesis, hypoxia, and mechanisms of immune evasion driven by the immunosuppressive tumor microenvironment (TME) [3]. The TME in HGGs supports tumor progression, immune evasion, and resistance to immunotherapies [4]. Key players in this immunosuppressive network include tumor-associated macrophages (TAMs), myeloid-derived suppressor cells (MDSCs), and regulatory T cells (Tregs), which impair the recruitment and activation of cytotoxic T lymphocytes and natural killer (NK) cells [5–7]. Our recent studies uncovered differences in the inflammatory status and cancer immunity within the TME of two different HGG RCAS murine models: one with an immunosuppressive TME (PDGFb) [8] and another characterized by a robust pro-inflammatory response, although associated with infiltration of immunosuppressive myeloid cells (BRAF V600E) [9] (Fig. 1A, Supplementary Fig. 1). Understanding and overcoming the immune reprogramming in HGGs remains challenging and requires an in-depth investigation of the molecular and epigenetic mechanisms driving immunosuppression.

In this context, the role of long non-coding RNAs (lncRNAs) in reprogramming the immunophenotype of HGG remains poorly investigated [10]. LncRNAs are RNA molecules over 200 nucleotides long, non-coding for proteins. They are poorly conserved and localized in the nucleus or cytoplasm. They form complex 3D structures, such as single-strand loops, double-stranded stems, or bulges, to regulate gene expression through different mechanisms, including chromatin remodeling, transcriptional regulation, and signal transduction. In the nucleus, LncRNAs act as enhancer, chromatin-modifying recruiters or conformation, transcription regulators, and are also involved in pre-mRNA splicing. In the cytoplasm, they influence protein-protein interactions, mRNA stability, and translation [11, 12]. In melanoma, lung, breast, colorectal, pancreatic, and other solid and hematological tumors [13–15], lncRNAs have been implicated in promoting several cancer-related processes, including differentiation, metabolism, survival, proliferation, migration, invasion, metastasis, DNA rearrangement, and extracellular matrix-crosstalk. Additionally, they play critical roles in reprogramming the tumor immune microenvironment (TIME), influencing T cell trafficking (H19), MDSC differentiation (PVT1, RUNXOR, Lnc57Rik), Treg-mediated immunosuppression (SNHG16), and promoting immune cell-cancer cell interactions (LNMAT1, JHDM1D-AS1). These findings suggest their involvement in cancer immune evasion mechanisms [10, 16, 17].

In this study, we investigated the correlation between specific lncRNA expression patterns and the immunosuppressive or pro-inflammatory milieu in HGGs. We identified a distinct lncRNA signature potentially associated with the high-grade glioma immunosuppressive TME. Given the unique immunological challenges posed by high-grade gliomas, understanding the role of lncRNAs in immune regulation could provide valuable insights into overcoming immune evasion and addressing current immunotherapy limitations, ultimately improving outcomes for patients with HGG.

## Results

### Analysis of TIME from different glioma models revealed distinct lncRNA signatures

To test our hypothesis, we used the immunocompetent, transgenic HGG RCAS t-va murine model (NTV-a: Ink4a + /−, Arf + /−, PTEN + /fl, LSL-Luc). Pups (0-2 days old) were injected with chicken fibroblast DF1 cells expressing RCAS plasmids designed to release retroviruses to drive the expression of PDGFb, BRAF V600E, and CRE in Tv-a-positive neural progenitors (Fig. 1A) [18, 19]. In these mice, gliomagenesis was driven by ectopic expression of oncogenic drivers (PDGFb or BRAF V600E) in a background heterozygous for PTEN, P14 and P16. The PDGFb model mimics transforming oligodendrogliomas in AYA patients, while the BRAF V600E model reflects transforming xanthoastrocytoma in pediatric patients [2, 9, 20]. Using these HGG murine models, we recently reported differences in inflammatory status and cancer immunity in the TME. The PDGFb mutation, which is frequent in AYA patients, is associated with an immunosuppressive TME [8]. In contrast, the BRAF V600E mutation, common in pediatric gliomas, is associated with a pro-inflammatory and dysregulated TME, characterized by infiltration of immunosuppressive myeloid cells and exhausted cytotoxic T cells together with pro-inflammatory immune cells [9] (Fig. 1A, Supplementary Fig. 1). We isolated tumor-infiltrating immune cells from HGG animals and analyzed their transcriptomes by bulk RNA sequencing (bulkRNAseq), comparing these profiles to immune cells isolated from age-matched tumor-free controls (Fig. 1B, C). Unsupervised hierarchical clustering of lncRNAs identified 1,063 significantly differentially expressed genes in PDGFb RCAS mice (516 upregulated and 547 downregulated, GSE288345; Fig. 1B, Supplementary Tables 1, 2), and 604 in BRAF V600E RCAS mice (216 upregulated and 388 downregulated, GSE252367; Fig. 1C, Supplementary Tables 1, 2). Further data processing, which excluded low-count transcripts, generated a more stringent list of differentially expressed lncRNAs: 255 for PDGFb, and 154 for BRAF V600E (Fig. 1D, Supplementary Table 1). A Venn diagram showed that 132 lncRNAs were differentially expressed in PDGFb, 31 in BRAF V600E, and 123 shared between the two models (Fig. 1D, Supplementary Table 1). Of these, 14 lncRNAs have already been reported in literature for their roles in modulating immune phenotypes in cancer (Table 1). Notably, MEG3, MIAT, Malat1, SNHG20, SNHG12, Ftx, Pvt1, Mir9-3hg expression in cancer immune cells was associated with an immunosuppressive phenotype, while H19, SNHG6, Trp53cor1, MiR17hg and MiR142hg were linked to a pro-inflammatory phenotype in cancer. The immunoregulatory mechanisms of Neat1 reported in literature (Table 1) are controversial. In immune cells from the PDGFb HGG model, the lncRNAs MEG3, MIAT, MiR9-3hg, Malat1, Ftx and SNHG20 were upregulated, while Pvt1, H19, Neat1, SNHG6, SNHG12, Trp53cor1 (lincRNA-p21), MiR17hg and Mir142hg were downregulated (Fig. 1E, Supplementary Fig. 2A). Interestingly, the lncRNA profile of immune cells from the BRAF V600E HGG model showed similar expression patterns of MALAT1, SNHG20 and Trp53cor1 compared to the PDGFb HGG model. However, the expression levels of Neat1, Mir142hg, Pvt1, Mir17hg, SNHG12 and Meg3 showed opposite trends (Fig. 1E, Supplementary Fig. 2B, C), which may correlate with the distinct inflammatory profiles and immunophenotypes of the different glioma models analyzed. Taken together, these findings suggest that specific immunomodulatory lncRNAs may actively contribute to shaping the glioma TIME (Fig. 1F).

**Table 1:** Summary of lncRNAs and their roles in the TIME of HGG RCAS models.

lncRNA	PDGFb	BRAF V600E	Cell localization	Role in cancer immune cells	Reference
	(Fc)	(Fc)			(PMID)
**Meg3**	36.27	0.04	nuclear and cytoplasmic	Negative correlation with infiltrating lymphocytes B, T (CD8+, CD4 + ), and myeloid cells (macrophages, neutrophils, dendritic cells) infiltration in low-grade gliomas.	34220951
**MIAT**	78.48		nuclear and cytoplasmic	Upregulated in Tregs, PDCD1 + CD8+ and GZMK + CD8 + T cells.	33221703
**Mir9-3hg**	11.44		nuclear	Promotes M2 polarization, and G-MDSC recruitment	32910548
					38145323
**Malat1**	1.64	2.16	nuclear	Promotes infiltration of MDSCs, TAMs, and inhibitory activity on effector T cells	37603945
					34295338
					37637063
**Ftx**	3.9		nuclear	Negative correlation with infiltrating CD8^+^ T-cells	32419983
					36359790
**SNHG20**	1.52	1.69	cytoplasmic	Promotes M2 polarization and cellular reprogramming	34970275
					35506150
**SNHG12**	0.38	1.78	cytoplasmic	Promotes M2 polarization	32927431
					37006268
					35658874
**SNHG6**	0.42		cytoplasmic	Negative correlation with infiltration of Neutrophils, DCs and macrophages	37006268
					34734036
					34794493
**Pvt1**	0.65	2.39	cytoplasmic	Overexpressed in MDSCs, promotes infiltration of G-MDSCs	30925926
					31404149
**Trp53Cor1**	0.29	0.38	nuclear	Tumor suppressor regulated by p53 pathway, reverses M2 polarization	32062693
**(lincRNA-p21)**					
**H19**	0.21		nuclear and cytoplasmic	Positive correlation with infiltrating CD4 + T, CD8 + T, B, DCs, neutrophils and macrophages	35810257
					37637063
**Neat1**	0.14	5.01	nuclear	Promotes inflammatory response, infiltration of M1-macrophage, influences the TIME. Regulates infiltration and antitumor activity of cytotoxic T cells, and controls M2 macrophage polarization.	37053197
					31731055
**Mir17hg**	0.15	1.81	nuclear	Prevent Tregs differentiation	21972292
					29499238
**Mir142hg**	0.11	2.54	nuclear	Controls differentiation and function of lymphocytes T, B, and myeloid cells (macrophages and dendritic cells)	38201290
					23809164

Expression levels, cellular localization, and roles of specific immunoregulatory lncRNAs in the tumor immune microenvironment (TIME) of RCAS-PDGFb (“cold” TIME) and RCAS-BRAF V600E (“hot” TIME) HGG models. Expression in RCAS HGG models is reported as fold change (Fc) relative to immune cells isolated from the brains of age-matched, no-tumor control mice. The roles of each lncRNA in cancer-related immune cells are summarized from the literature. References (PMID) are provided for each lncRNA.

### Differential expression of immunomodulatory lncRNAs in glioma-associated immune cells

**Fig. 2:**
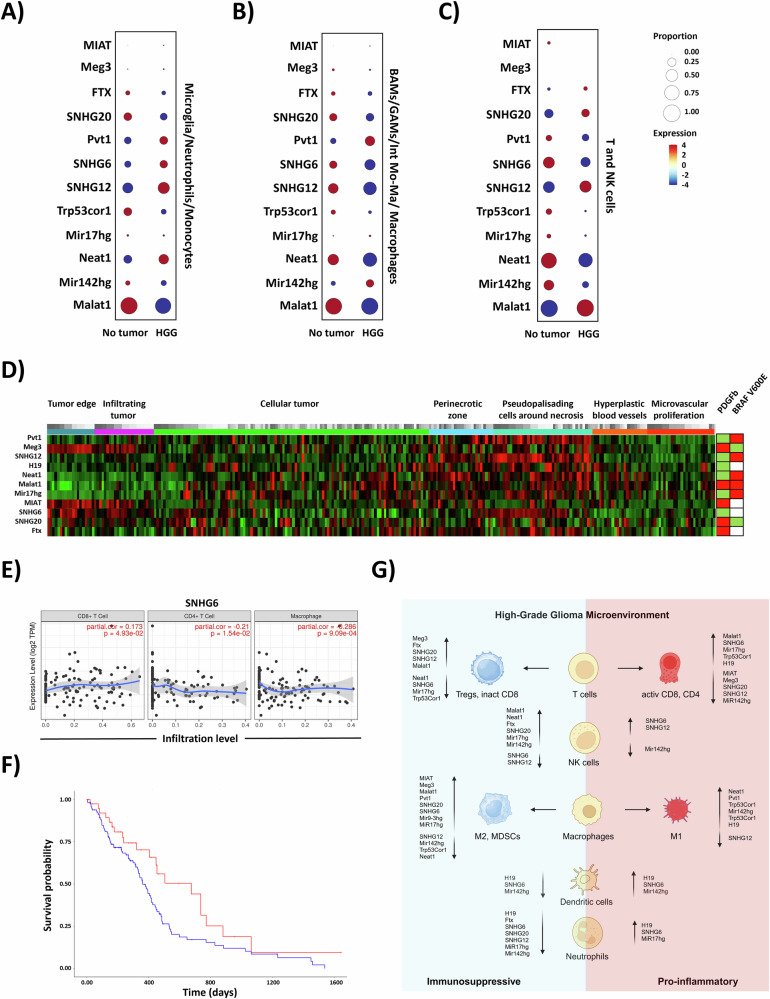
Expression of immunoregulatory lncRNAs in glioma-infiltrating immune cells. Bubble plots showing the proportion and expression levels of specific lncRNAs in: (**A**) pro-inflammatory myeloid cells, **B** immunosuppressive myeloid cells, and (**C**) T and NK cells. **A–C** RCAS-PDGFb transcriptome analyzed by scRNAseq. **D** Spatial distribution of immunoregulatory lncRNAs by laser microdissection of immune cell populations in the TME of 41 GBM patients. **E** Scatterplots showing the correlation between specific lncRNAs and immune infiltration levels in GBM patients. Each plot includes a fitted regression line (), partial correlation coefficients (), and *p*-values. **F** Kaplan-Meier survival curves comparing the survival of glioblastoma multiforme (GBM) patients with high (red, *n* = 39) versus low (blue, *n* = 113) expression of the lncRNA SNHG6, evaluated from the TGCA database (log-rank test, *p* = 0.03). **G** Illustration summarizing the main findings of the study (biorender.com).

Using scRNAseq analysis, we recently showed the immunosuppressive reprogramming of glioma-associated immune cells within the TME during tumor progression (GSE221440). This analysis identified four immunosuppressive myeloid subpopulations (glioma associated macrophages [GAMs], border associated macrophages [BAMs], intermediate monocytes/macrophages [Int Mo/Mac], and macrophages), and three pro-inflammatory myeloid populations (monocytes, neutrophils, and microglia). Among the immunosuppressive subtypes, BAMs and Int Mo/Mac showed increased expression of genes associated with bone marrow-derived myeloid cells (BMDMs), while GAMs and macrophages showed enrichment in tumor-associated macrophage (TAM) gene signature. Additionally, HGG mice were characterized by poor infiltration of T and NK cells, most of which were exhausted [8]. In both HGG murine models (PDGFb and BRAF V600E), myeloid cells were the most abundant immune cell types in the TME, reflecting the immune landscape observed in glioma patients [8, 9]. Using the immunosuppressive scRNAseq signature from RCAS-PDGFb tumor-infiltrating immune cells, we analyzed the expression of immunomodulatory lncRNAs (Fig. 2A–C, Supplementary Fig. 3). The scRNAseq analysis revealed that neutrophils within the immunosuppressive HGG TME were associated with the majority of downregulated immunoregulatory lncRNAs. Neutrophils have been recently implicated in cancer-related immunosuppressive mechanisms. Upon infiltration into the tumor microenvironment, they can differentiate into immunosuppressive tumor-associated neutrophils (TANs), promoting cancer progression, angiogenesis, and tumor cell invasion [7]. However, the mechanisms driving the reprogramming of neutrophils into TANs remain poorly investigated.

The analysis of immunomodulatory lncRNA expression in the HGG-PDGFb immunosuppressive TIME identified Meg3 as upregulated in immunosuppressive myeloid cells and in microglia cells. Meg3 was negatively correlated with the infiltration of naïve myeloid and T cells. Interestingly, MIAT, typically upregulated in Tregs and exhausted T cells, showed opposite trends: it was upregulated in myeloid cells but downregulated in T cells. Mir9-3hg, which promotes M2 polarization and MDSCs, was upregulated in immune cells analyzed by bulk RNAseq but not detectable in the tumor immune cells analyzed by scRNAseq. Ftx, known to negatively correlate with cytotoxic T cell infiltration, was downregulated in neutrophils but upregulated in T and NK cells. Malat1, a key lncRNA in cancer and immune cell function that inhibits cytotoxic T cells activation and promote immunosuppressive myeloid cells infiltration, was upregulated in T and NK cells and immunosuppressive BAMs but downregulated in immunosuppressive GAMs, int Mo/Mac and Macrophages (Supplementary Fig. 3B). The lncRNAs SNHG12 and SNHG20, both involved in promoting M2 polarization, showed distinct expression patterns in the RCAS-PDGFb model: SNHG12 was upregulated in T cells, microglia and BAMs, but downregulated in NK cells, neutrophils, GAMs, and macrophages, while SNHG20 was upregulated in NK cells, but downregulated in microglia, neutrophils, and Int Mo/Mac and macrophages. Additionally, in the RCAS-PDGFb TME SNHG12 was downregulated, while SNHG20 was upregulated. These findings highlight distinct roles for SNHG12 and SNHG20 in the HGG TIME. Pvt1, significantly expressed in MDSCs, was upregulated in microglia, BAMs and macrophages, but downregulated in immunosuppressive Int Mo/Mac and in GAMs, suggesting a potential role in myeloid cell infiltration in glioma. SNHG6, which has been reported in literature to inversely correlate with macrophages, neutrophils, and dendritic cells (DCs) infiltration in lung adenocarcinoma, was unexpectedly upregulated in microglia, BAMs, GAMs, and macrophages, but was downregulated in NK cells. Notably, analysis of the TCGA database for HGG patients demonstrated that lower expression of SNHG6 was significantly associated with worst overall survival (Fig. 2F, Supplementary Fig. 4B). Trp53cor1 (linc-p21), which participates in regulating the pro-inflammatory p53 pathway, was downregulated in both PDGFb and BRAF V600E signatures. Specifically, it was downregulated in T, NK, and myeloid cells except for microglia, where it was upregulated. This indicates a pivotal role for Trp53cor1 in TIME reprogramming. The role of Neat1 in regulating cancer immune cells infiltration and activation remains controversial. Although Neat1 has been associated with promoting inflammatory responses by regulating M1 macrophages and cytotoxic T cells, it has also been linked to M2 polarization. In the immunosuppressive glioma TIME, Neat1 was upregulated in NK cells but downregulated in immunosuppressive myeloid cells and pro-inflammatory T cells. Mir17hg, the precursor of the MiR17-92 clusters, which prevents CD4 + T cells differentiation into Tregs, was significantly downregulated in Neutrophils but upregulated in T cells and in immunosuppressive myeloid cells, suggesting additional involvement in the HGG TIME. Mir142hg, involved in controlling the differentiation and function of pro-inflammatory T, B, and myeloid cells, was upregulated in immunosuppressive myeloid cells and in NK cells, but downregulated in T cells and neutrophils.

The spatial distribution of the expression of these lncRNAs in glioblastoma (GBM) patients was analyzed using transcriptomic data generated by laser microdissection on 41 glioblastoma patients (Ivy GAP project [21]). Interestingly, the analysis revealed that Meg3, MIAT and SNHG20 are primarily expressed by cells localized at the tumor edge or infiltrating the tumor core. SNHG6 was expressed at the tumor edge and in the pseudopalisading regions around necrosis, but most lncRNAs (Pvt1, SNHG12, H19, Neat1, Malat1, Mir17hg and Ftx) were expressed around the necrotic tumor regions (Fig. 2D). Glioma-associated necrosis, a hallmark of high-grade gliomas, is linked to poor prognosis due to its role in immunosuppression and tumor progression [22]. To validate these findings, we analyzed correlations between lncRNA expression and immune cells infiltration in GBM patients using deconvolution analysis (TIMER [23]). This confirmed the expression of MiR17hg, SNHG6, H19 and Pvt1 in glioma-infiltrating immune cells, consistent with our murine models (Fig. 2E, Supplementary Fig. 4A). Taken together, these results suggest that specific lncRNAs may be involved in defining immune cell trajectories within the HGG TIME.

## Discussion

High-grade gliomas (HGGs) in pediatric, adolescent, and young adult (AYA) patients are characterized by aggressive progression and poor prognosis, largely driven by a highly immunosuppressive tumor microenvironment (TME) [4]. Our study underscores the critical roles of lncRNAs in shaping the immune landscape within the HGG TME, suggesting that lncRNAs could regulate immune cell infiltration and functionality, thereby contributing to tumor progression and resistance to therapy. The role of lncRNAs in cancer immunity is a rapidly emerging field. LncRNAs have been shown to either promote or inhibit the recruitment of tumor-infiltrating lymphocytes (TILs), which are crucial for anti-tumor immunity. Additionally, they regulate macrophage polarization within the TME, shifting them toward a pro-inflammatory (M1) or immunosuppressive (M2) phenotype. By modulating the balance between pro-inflammatory and immunosuppressive signals, lncRNAs significantly shape the immune landscape of tumors [16, 17]. However, only a limited number of recent studies have explored their role in reprogramming the TME to promote tumor progression and immune evasion [17, 24]. In addition, no studies to date have systematically investigated the role of lncRNAs in tumor-infiltrating immune cells within the context of immunosuppressive or pro-inflammatory glioma TIME in vivo.

Using RCAS/t-va murine models with PDGFb and BRAF V600E mutations, we identified distinct lncRNA signatures associated with the immunosuppressive and pro-inflammatory milieus of gliomas [8, 9]. These lncRNAs were linked to opposite immune profiles: PDGFb tumors predominantly exhibited an immunosuppressive TME [8], while BRAF V600E tumors displayed a dysregulated pro-inflammatory TME characterized by infiltration of activated or exhausted cytotoxic T cells, as well as pro-inflammatory or immunosuppressive myeloid cells [9] (Fig. 2G).

Key findings revealed that immune cells isolated from immunosuppressive HGG TME (PDGFb RCAS model) exhibited upregulation of Meg3, MIAT, Mir9-3hg, and Ftx, which are associated with MDSCs activation, and cytotoxic T cells inactivation. Meg3 was significantly downregulated in a pro-inflammatory HGG TME. On the other hand, lncRNAs linked to the activation of pro-inflammatory macrophages and cytotoxic effectors (Neat1, Mir17hg, Mir142hg) showed the opposite trend. The expressions of Malat1, Trp53Cor1, and SNHG20 were consistently correlated with immunosuppression in both RCAS models. Notably, Pvt1 and SNHG12 deviated from expected expression patterns based on the literature, when correlated with the inflammatory status of the TME. Further scRNAseq analysis of immunoregulatory lncRNAs in immune cells infiltrating the immunosuppressive glioma TME revealed that neutrophils exhibited downregulation of several immunoregulatory lncRNAs. These results can be relevant after recent studies showing the direct involvement of immunosuppressive tumor associated Neutrophils (TANs) in glioma progression [7]. Moreover, specific lncRNAs were differentially expressed among distinct subtypes of immunosuppressive myeloid cells, emphasizing their pivotal role in reprogramming myeloid cells. This context-dependent expression suggests the functional plasticity of lncRNAs. Spatial transcriptomic analysis in GBM patients further linked specific lncRNAs to necrotic regions and tumor edges, correlating their expression with immunosuppressive areas of the glioma TME. The correlation between lncRNAs expression and immunophenotype in glioma, along with the poor survival of glioma patients with reduced SNHG6 expression, were translationally relevant. However, the absence of a direct correlation between our full murine lncRNA signature and survival data in the TCGA cohort may be due to the extremely limited pediatric and AYA HGG samples. While our findings underscore the potential role of lncRNAs in shaping the immunosuppressive glioma TME, we acknowledge that their therapeutic relevance remains to be fully addressed. In vivo modulation of specific lncRNAs will be essential to understand their involvement in immune cell reprogramming and glioma progression. This can be achieved using antisense oligonucleotides (ASOs), small interfering RNAs (siRNAs), or CRISPR-based approaches. These technologies are already under evaluation in preclinical and clinical studies for targeting lncRNAs in other malignancies [25]. For instance, silencing lncRNAs such as MEG3, FTX, and MIAT in tumor-infiltrating myeloid or T cells, or enhancing expression of pro-inflammatory lncRNAs like NEAT1 or MIR17HG in T cells, may reduce immunosuppression, promote infiltration, and enhance activation of anti-tumor immunity.

Our study provides a foundational list of immunomodulatory lncRNAs that could be leveraged to reprogram the glioma immune landscape and potentiate responses to immunotherapies.

Understanding how lncRNAs contribute to immune evasion and TME remodeling could pave the way for the development of novel combinatorial immunotherapies targeting lncRNA expression and blocking immune checkpoints in the glioma TME, to enhance cytotoxic immune cell recruitment and activation, ultimately improving outcomes for HGG patients.

## Methods

All methods are described in the Supplementary Material.

## Supplementary information

Supplementary methods

Supplementary figures

Supplementary Table 1

Supplementary Table 2

## Data Availability

Upon request, the corresponding authors will grant access to all data for the scientific community.
