# Supplementary material for: Identification of an immunomodulatory lncRNA signature associated with immune cell reprogramming in high-grade glioma
Source: Cancer Gene Ther. 2025 Jun 17;32(7):778–84. doi: 10.1038/s41417-025-00919-3 (PMC12277174; doi:10.1038/s41417-025-00919-3)
Supplement: Supplementary file 1 — Supplementary methods [file 41417_2025_919_MOESM1_ESM.pdf]

Supplementary methods.

Mouse model. Gliomagenesis in vivo was induced using the NTV-a model (Ink4a$^{+/-}$, Arf$^{+/-}$, PTEN$^{+/fl}$, LSL-Luc) and the RCAS system[8,9, 18-20]. Pups aged 0-2 days were injected using 1µL of PBS (Gibco) containing 50,000 DF1-PDGFb and 50,000 DF1-CRE cells. DF1 cells (ATCC) were tested for mycoplasma contamination periodically, and prior to experimental use. The RCAS system is designed to deliver retrovirus into the bregma of newborn pups, transducing neural stem cells expressing the T-va receptor (n=3, 4). Age-matched, genetically identical animals without tumors were used as negative controls (n=3). Animals with premature development of hydrocephalus or early signs of decline due to tumor progression were excluded from the study. Similarly, tumor-negative animals, as determined by IVIS imaging, were excluded from the tumor group analysis. Randomization of mice was not required for this study. Animal studies were approved by Nationwide Children's Hospital Institutional Animal Care and Use Committee (protocol # AR19-00146).

Isolation of immune cells from the glioma tumor microenvironment. Following euthanasia of HGG animals, brains were extracted, and the right hemisphere was isolated, transferred to a six-well plate, and kept in PBS on ice. To generate a single-cell suspension, brains were mechanically dissociated using a 70µM strainer. Cells were washed in PBS, stratified using 25% Percoll (GE Healthcare), and centrifuged at 500g for 20 minutes at 18°C. The resulting immune cell pellet was washed twice with PBS and processed for further analysis (n=3, 4).

RNA sequencing

Bulk RNA sequencing. Total RNA was extracted using the RNeasy plus mini kit (Qiagen) and the RNA clean-up and Concentration kit (Norgen). RNA quality was assessed using the Bioanalyzer RNA Chip (Agilent), and the quantification was measured with the Qubit RNA High Sensitivity Assay (Thermo Fisher Scientific). Library preparation, sequencing, data alignment and normalization were performed by the Genomic Core Facility of the Nationwide Children's Hospital (Columbus, Ohio), as previously described[9] (n=3, 4). Statistical significance was calculated using the student's *t*-test. Clustering analysis and heatmap generation were performed using Cluster 3.0, and Java Treeview. Volcano plots were generated using GraphPad (Prism), and Venn diagrams were created with the Bioinformatics & Evolutionary Genomics webtool (bioinformatics.psb.ugent.be/webtools/Venn/). Gene set enrichment analysis was performed using GSEA (www.gsea-msigdb.org/gsea). Normalized counts and raw files are publicly available in the GEO data repository (accession numbers GSE288345 and GSE252367).

Single cell RNA sequencing (scRNAseq). Samples were processed and analyzed as previously described[8]. Bubble plots were generated using R software, and the database is publicly available in the GEO data repository (accession number GSE221440).

Expression of lncRNAs in GBM patients.

Ivy-GAP database. To analyze the spatial distribution and expression of a specific cohort of immunomodulatory lncRNAs in human HGG patients, we used the Ivy Glioblastoma Atlas Project (Ivy-GAP, http://glioblastoma.alleninstitute.org/) webtool, which contains the RNA-seq profiles of 270 laser-microdissected samples from 41 GBM tumors. Tumor microenvironment (TME)

locations analyzed include tumor edge, Infiltrating tumor, cellular tumor, perinecrotic zone, pseudopalisading cells around necrosis, hyperplastic blood vessels, and microvascular proliferation.

Timer database. To evaluate correlations of specific immunomodulatory lncRNAs and tumor-infiltrating immune cells in GBM patients, we used the TIMER database (cistrome.shinyapps.io/timer/). Correlation plots include statistical significance and correlation coefficient (Spearman's rho value).

TGCA database. To assess the prognostic impact of specific immunomodulatory lncRNAs in glioma patients, we interrogated the TGCA database using the UALCAN (ualcan.path.uab.edu) and Gepia2 (gepia2.cancer-pku.cn) online tools. Kaplan Meier survival curves were generated to visualize the association between lncRNA expression and glioma patient outcomes.

Statistical analysis: The results were generated from at least three independent observations. Statistical significance (p-values <0.05) was assessed using an unpaired two-tailed Student's t-test and long-rank test using GraphPad (Prism).
