# Supplementary material for: Identification of an immunomodulatory lncRNA signature associated with immune cell reprogramming in high-grade glioma
Source: Cancer Gene Ther. 2025 Jun 17;32(7):778–84. doi: 10.1038/s41417-025-00919-3 (PMC12277174; doi:10.1038/s41417-025-00919-3)
Supplement: Supplementary file 2 — Supplementary figures [file 41417_2025_919_MOESM2_ESM.pdf]

A)

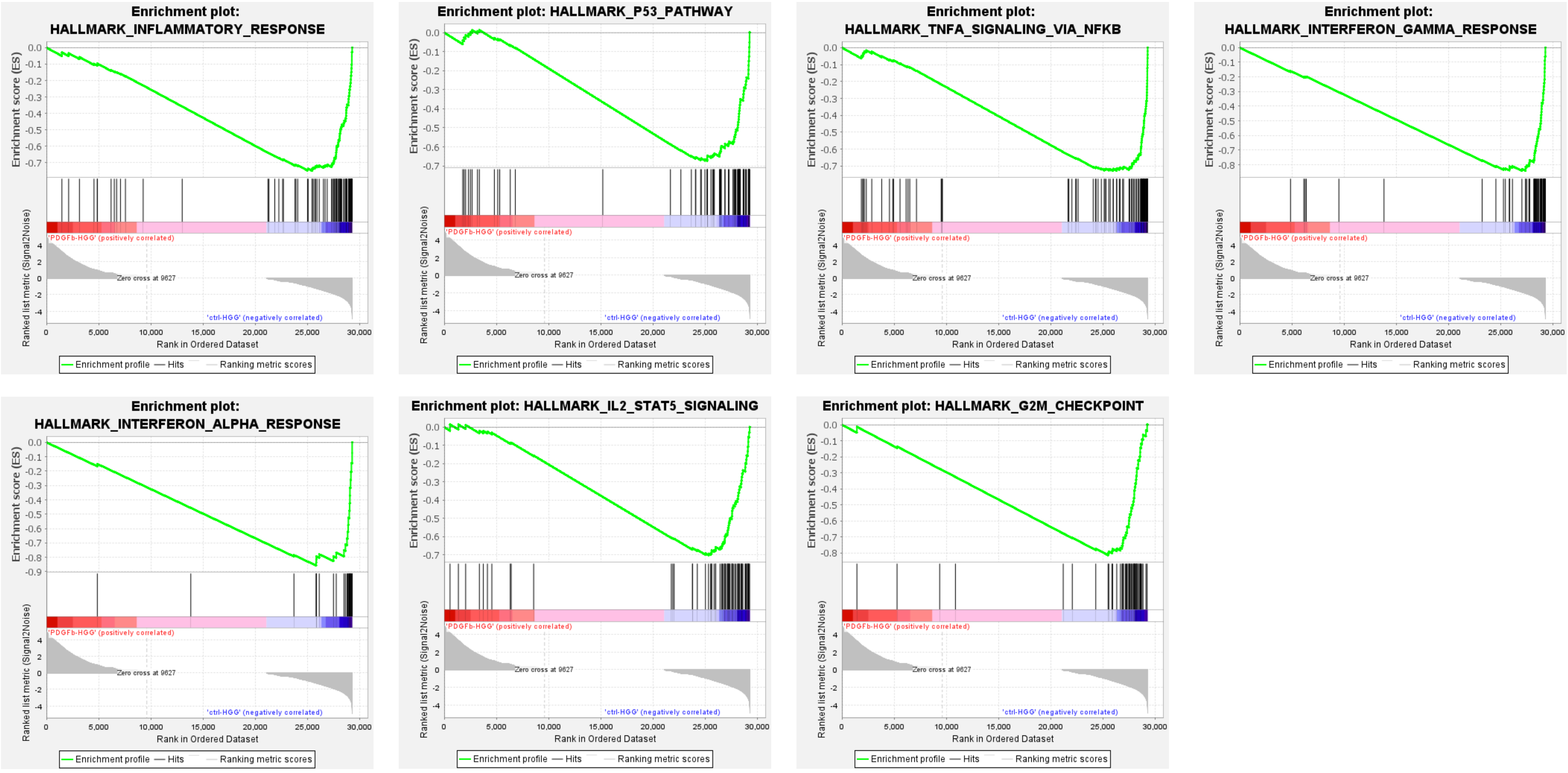

B)

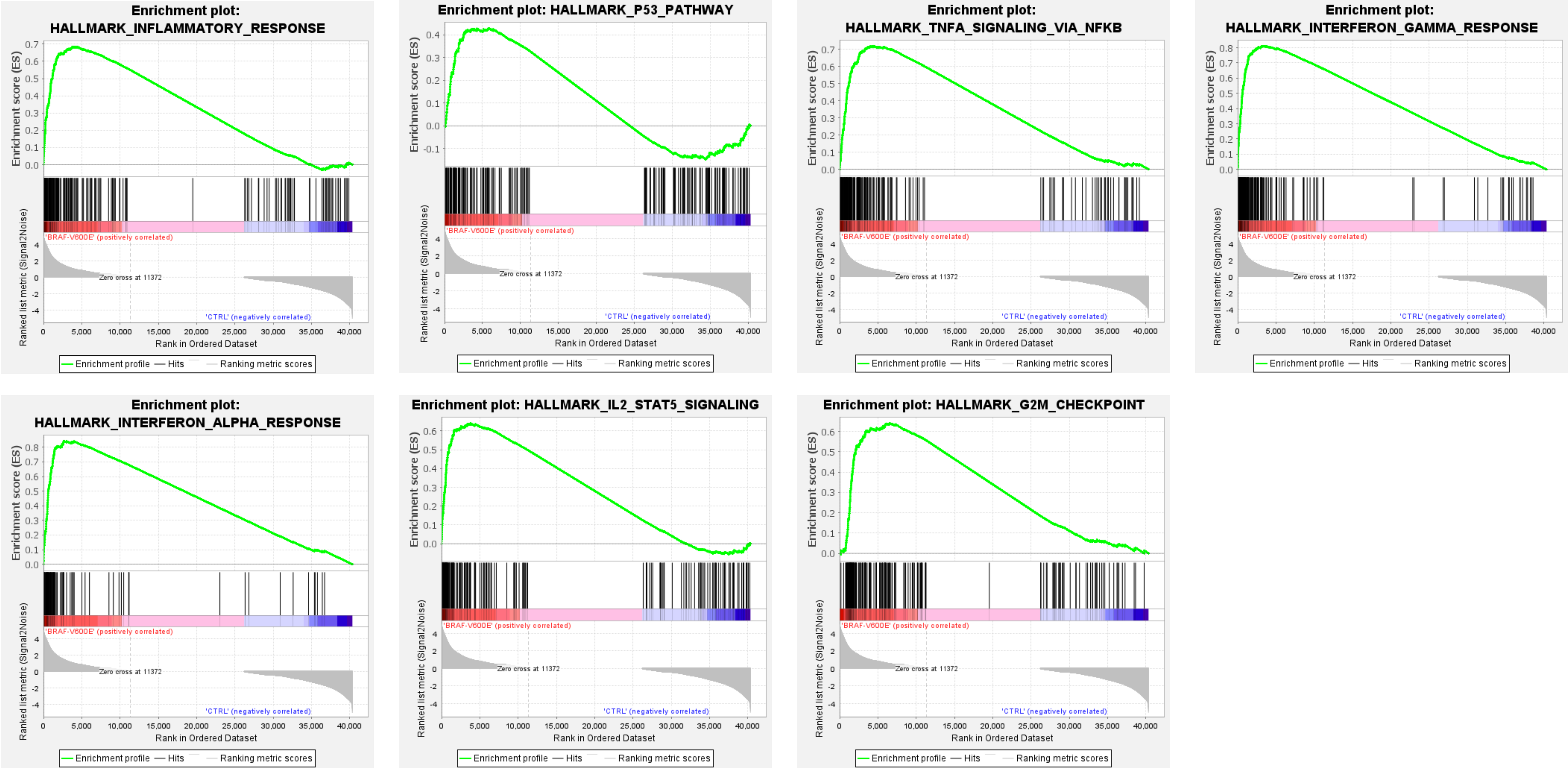

Supplementary Figure 1. Gene Set Enrichment Analysis (GSEA) of pro-inflammatory pathways in immune cells of the HGG TIME. A) GSEA analysis conducted on immune cells isolated from the TME of the immunosuppressive RCAS-PDGFb model (n=4) and compared to immune cells from age-matched no-tumor control animals (n=3). **B)** GSEA analysis performed on immune cells isolated from the TME of the pro-inflammatory RCAS-BRAF V600E model (n=3) and compared to immune cells from age-matched no-tumor controls (n=3).

A)

Symbol	Class	NO TUMOR CTLRs			PDGFb				log2(Fc)	*P*-value
MIAT	lincRNA	243	228	282	17673	17496	21435	22194	6.29	4.222E-05
Meg3	lincRNA	7279	6758	9536	292593	258168	275917	313254	5.18	5.925E-06
Mir9-3hg	lincRNA	101	78	66	929	918	951	939	3.52	1.032E-08
Ftx	lincRNA	354	516	603	1731	1957	2044	1931	1.96	2.959E-05
Malat1	lincRNA	310558	352297	449650	605174	575806	630759	622970	0.71	0.001417
SNHG20	lincRNA	258	338	370	485	506	492	469	0.60	0.002388
Pvt1	lincRNA	362	608	589	313	330	346	370	-0.61	0.044921
SNHG6	lincRNA	141	252	245	102	90	101	66	-1.24	0.011555
SNHG12	lincRNA	437	575	538	228	180	178	198	-1.40	0.000343
Trp53cor1	lincRNA	664	514	437	185	143	143	143	-1.81	0.001093
H19	lincRNA	129	163	159	47	52	8	20	-2.24	0.000591
Mir17hg	lincRNA	100	176	200	17	17	25	36	-2.74	0.003368
Neat1	lincRNA	10057	13711	20662	2040	2135	2239	2003	-2.82	0.004549
Mir142hg	lincRNA	1838	1819	2224	214	202	212	209	-3.23	1.816E-05

B)

Symbol	Class	NO TUMOR CTLRs			BRAF V600E			log2(Fc)	*P*-value
Neat1	lincRNA	2338	2110	1872	11612	10566	9478	2.32	0.00018
Mir142hg	lincRNA	487	418	486	1364	1238	937	1.35	0.005114
Pvt1	lincRNA	277	222	317	703	621	629	1.26	0.000564
Malat1	lincRNA	80015	75836	79729	178133	189925	140877	1.11	0.003569
Mir17hg	lincRNA	30	34	48	70	63	70	0.86	0.006929
SNHG12	lincRNA	254	259	349	496	570	470	0.83	0.006413
SNHG20	lincRNA	82	74	66	121	122	133	0.76	0.001031
Trp53cor1	lincRNA	365	321	340	117	170	99	-1.41	0.001008
Meg3	lincRNA	6793	7043	5068	106	315	356	-4.60	0.000643

C)

		log2(Fc)	
Symbol	Class	BRAF V600E	PDGFb
MIAT	lincRNA	6.29	
Meg3	lincRNA	5.18	-4.60
Mir9-3hg	lincRNA	3.52	
Ftx	lincRNA	1.96	
Malat1	lincRNA	0.71	1.11
SNHG20	lincRNA	0.60	0.76
Pvt1	lincRNA	-0.61	1.26
SNHG6	lincRNA	-1.24	
SNHG12	lincRNA	-1.40	0.83
Trp53cor1	lincRNA	-1.81	-1.41
H19	lincRNA	-2.24	
Mir17hg	lincRNA	-2.74	0.86
Neat1	lincRNA	-2.82	2.32
Mir142hg	lincRNA	-3.23	1.35

Supplementary Figure 2. Differential expression of lncRNAs in different glioma TIMEs. Assessment by RNA sequencing of the expression of specific immunoregulatory lncRNAs in tumor-infiltrating immune cells isolated from: **A)** HGG RCAS-PDGFb (n=4) compared with no-tumor controls (n=3), and **B)** HGG RCAS-BRAF V600E (n=3) compared with no-tumor controls (n=3). Upregulated lncRNAs compared to controls are shown in **red**, while downregulated lncRNAs are shown in **blue**. **C)** Direct comparison between the lncRNA expression in tumor-infiltrating immune cells isolated from RCAS-BRAF V600E and RCAS-PDGFb compared with no-tumor controls. Data are expressed as normalized counts, fold change (log2 Fc), and *P*-values.

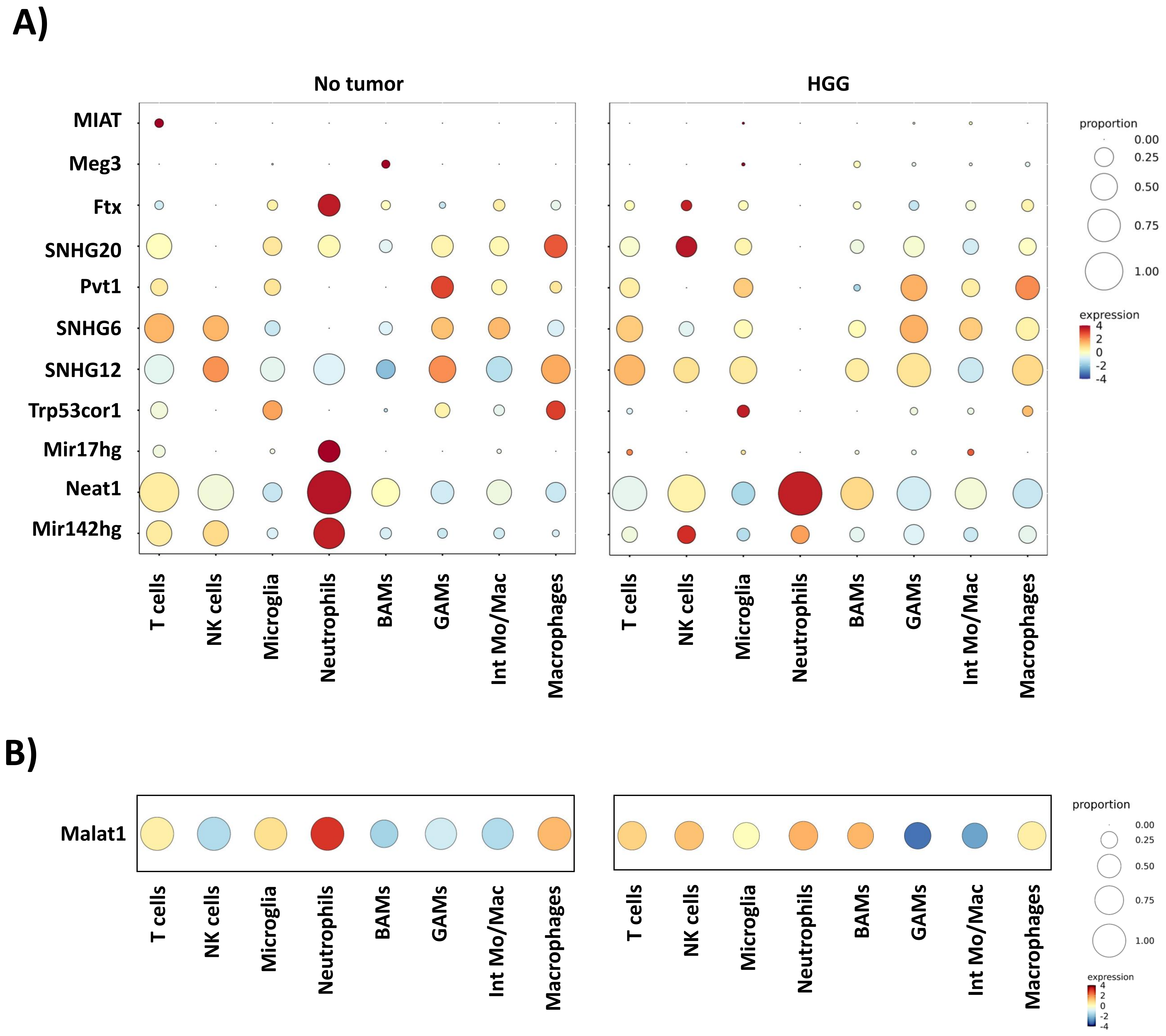

Supplementary Figure 3. Expression and immune infiltration analysis of lncRNAs. (A) Bubble plots showing the proportion and expression levels of specific lncRNAs across different immune cell types. **(B)** The Malat1 bubble plot was analyzed separately due to its overexpression compared to other lncRNAs.

A)

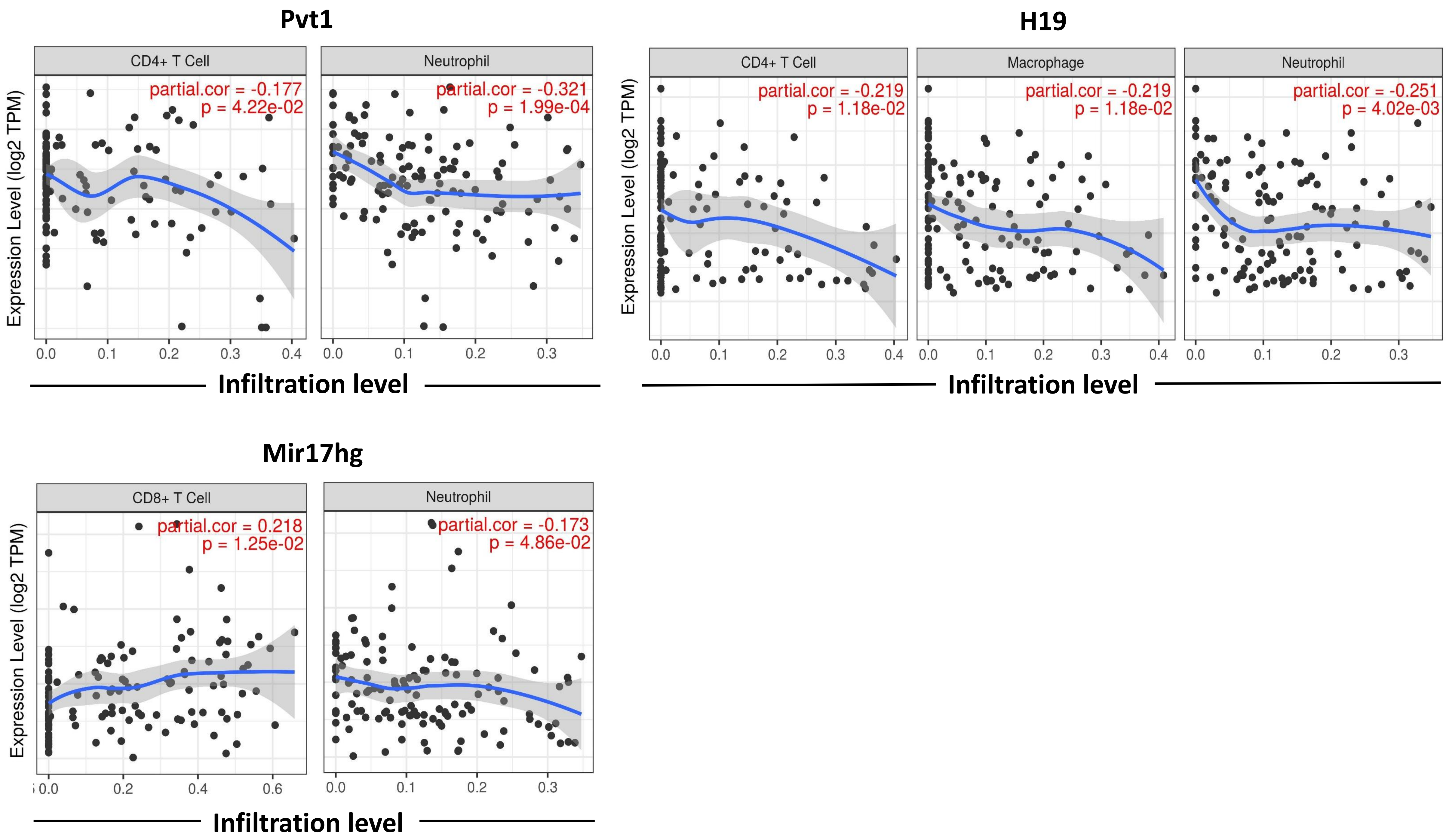

B)

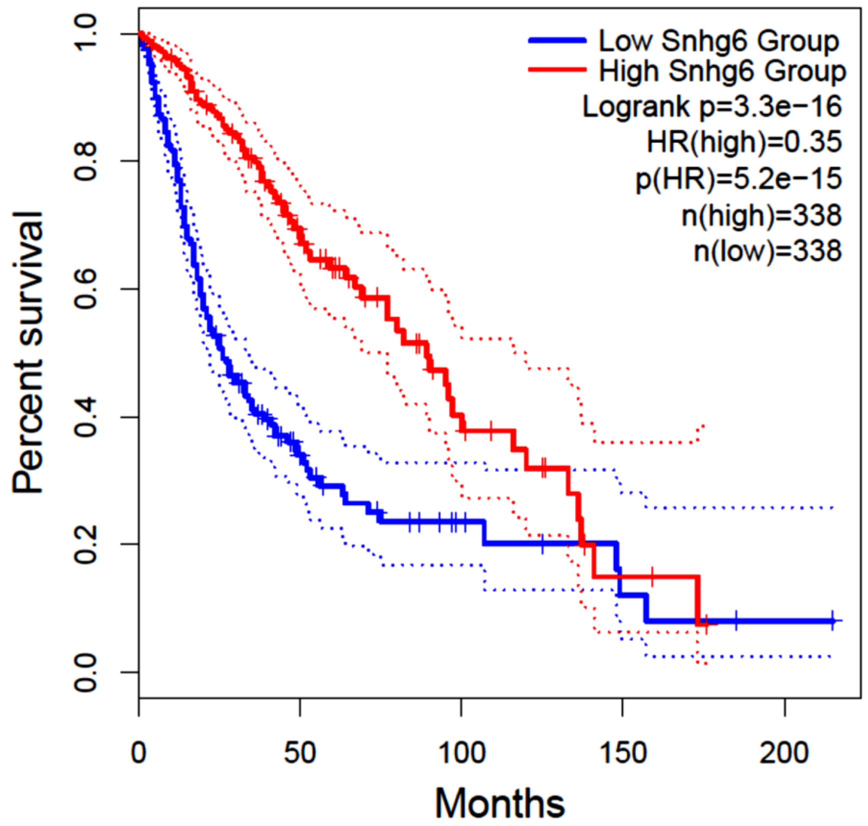

Supplementary Figure 4. Translational relevance of immune-related lncRNAs. (A) Correlation scatterplots showing the relationship between Pvt1, H19 and Mir17hg expression and immune cell infiltration in GBM patients. Each scatterplot includes a fitted regression line (**blue**), partial correlation coefficients (**red text**), and associated *p*-values. **(B)** Kaplan–Meier survival curve showing the overall survival of glioma patients stratified by SNHG6 expression levels, using TCGA glioma datasets (LGG + GBM) via GEPIA2. Statistical significance was assessed using the log-rank test.

Supplementary Table Legends

Supplementary Table 1. List of the lncRNAs significantly differentially expressed in the TIME of HGG RCAS models. The table list lncRNAs that are differentially expressed specifically in the TIME of HGG RCAS-PDGFb, shared between both HGG RCAS-PDGFb and HGG RCAS-BRAF V600E, and exclusively in HGG RCAS-BRAF V600E.

Supplementary Table 2. LncRNA signature in the tumor-infiltrating immune cells from HGG RCAS-PDGFb and HGG RCAS-BRAF V600E mice, compared to immune cells from brains of age-matched no-tumor controls.
